# Fluorescence activated cell sorting and fermentation analysis to study rumen microbiome responses to administered live microbials and yeast cell wall derived prebiotics

**DOI:** 10.3389/fmicb.2022.1020250

**Published:** 2023-03-03

**Authors:** Leeann Klassen, Greta Reintjes, Meiying Li, Long Jin, Carolyn Amundsen, Xiaohui Xing, Lharbi Dridi, Bastien Castagner, Trevor W. Alexander, D. Wade Abbott

**Affiliations:** ^1^Lethbridge Research and Development Centre, Agriculture and Agri-Food Canada, Lethbridge, AB, Canada; ^2^Department of Pharmacology & Therapeutics, Max Planck Institute for Marine Microbiology, Bremen, Germany; ^3^College of Food Science, South China Agricultural University, Guangzhou, China; ^4^Faculty of Medicine and Health Sciences, McGill University, Montreal, QC, Canada

**Keywords:** rumen, microbiome, *Bacteroides*, mannan, yeast cell wall, fermentation, glycomics

## Abstract

Rapid dietary changes, such as switching from high-forage to high-grain diets, can modify the rumen microbiome and initiate gastrointestinal distress, such as bloating. In such cases, feed additives, including prebiotics and live microbials, can be used to mitigate these negative consequences. Bio-Mos® is a carbohydrate-based prebiotic derived from yeast cells that is reported to increase livestock performance. Here, the responses of rumen bacterial cells to Bio-Mos® were quantified, sorted by flow cytometry using fluorescently-labeled yeast mannan, and taxonomically characterized using fluorescence *in situ* hybridization and 16S rRNA sequencing. Further, to evaluate the effects of bovine-adapted *Bacteroides thetaiotaomicron* administration as a live microbial with and without Bio-Mos® supplementation, we analyzed microbial fermentation products, changes to carbohydrate profiles, and shifts in microbial composition of an *in vitro* rumen community. Bio-Mos® was shown to be an effective prebiotic that significantly altered microbial diversity, composition, and fermentation; while addition of *B. thetaiotaomicron* had no effect on community composition and resulted in fewer significant changes to microbial fermentation. When combined with Bio-Mos®, there were notable, although not significant, changes to major bacterial taxa, along with increased significant changes in fermentation end products. These data suggest a synergistic effect is elicited by combining Bio-Mos® and *B. thetaiotaomicron*. This protocol provides a new *in vitro* methodology that could be extended to evaluate prebiotics and probiotics in more complex artificial rumen systems and live animals.

## 1. Introduction

Concentrate-based diets are often used to finish beef cattle by increasing total weight gain and fat composition (Bidner et al., 1981; Williams et al., 1983). Concentrates, such as corn and wheat grain, are rich in carbohydrates that are rapidly fermented by the gut microbiome. High concentrate diets are also associated with a lower diversity of operational taxonomic units (OTUs; Petri et al., 2013), ruminal acidosis (Nagaraja and Titgemeyer, 2007), liver abscesses (Nagaraja and Chengappa, 1998), and endotoxins (Nagaraja and Titgemeyer, 2007; Dong et al., 2011; Zebeli and Metzler-Zebeli, 2012); often these ailments are treated using antimicrobials. Heightened concern by consumers and policy makers over the emergence of antimicrobial resistance in bacteria (Auffret et al., 2017; WHO, 2019) and adoption of a one-health approach have led to curtailed use of antimicrobials in beef production, practices which will benefit from the development of effective alternatives to antimicrobials.

Administration of carbohydrates that act as prebiotics, to selectively increase the abundance of beneficial bacteria; or probiotics to stimulate improvements in gut health, have been shown to promote normal ruminal pH (Vyas et al., 2014), increase volatile fatty acid (VFA) production (Lao et al., 2020) and microbial diversity (Pinloche et al., 2013), and improve animal performance (Heinrichs et al., 2003). Understanding the mechanism(s) by which prebiotics and probiotics contribute to animal health, ruminal fermentation, and digestive processes will be pivotal to their adoption by beef producers. Further, there is little research combining prebiotics and probiotics, otherwise known as ‘synbiotics’, in a rumen environment to elicit a synergistic effect on host health and digestion (Yasuda et al., 2007; Markowiak and Śliżewska, 2018).

α-Mannooligosaccharides (MOS) are structurally complex fragments of yeast mannan (YM; Jones et al., 2020) and one of the most widely studied and used prebiotics for cattle. MOS have been used to increase carcass weight and alter VFA production (Ghosh and Mehla, 2012), decrease inflammation (Garcia Diaz et al., 2018), prevent acidosis (Vyas et al., 2014), and prime the immune system (Broadway et al., 2015). Bio-Mos® is a commercial livestock prebiotic derived from the cell walls of *Saccharomyces cerevisiae*, and is composed of highly branched α-mannans (Cuskin et al., 2015), along with β-glucans and chitin (Abbott et al., 2015). Here, we investigate the probiotic potential of *Bacteroides thetaiotaomicron* (*B. theta*) in rumen microbial communities. *B. theta* is a member of the phylum Bacteroidetes, which is one of the dominant rumen phyla (Petri et al., 2013; Henderson et al., 2015) known for possessing an abundance of polysaccharide utilization loci (PULs) that enable the uptake and metabolism of a wide range of complex carbohydrates (Grondin et al., 2017) and for having a higher association with feed particles in the rumen relative to Firmicutes members (Pinnell et al., 2022). Therefore, administration could potentially lead to improved intestinal health through competitive exclusion of pathogens and improved digestion of dietary fiber. Additionally, *B. theta* encodes three YM PULs that have all the tools to sense, import, and saccharify YM (Cuskin et al., 2015; Klassen et al., 2021), suggesting it would be a good candidate to combine with Bio-Mos® as a synbiotic. Here, we use fluorescently labeled YM (FLA-YM), fluorescence activated cell sorting (FACS), and 16S rRNA sequencing to visualize, sort, and identify bacteria that interact with YM, respectively. Further, we evaluate rumen communities supplemented with Bio-Mos® and/or *B. theta* strains MD33 and MD40, which were previously isolated from the bovine feces and shown to display different foraging behaviors on YM (Klassen et al., 2021).

## 2. Results

### 2.1. Microscopy and fluorescence *in situ* hybridization

**Figure 1:**
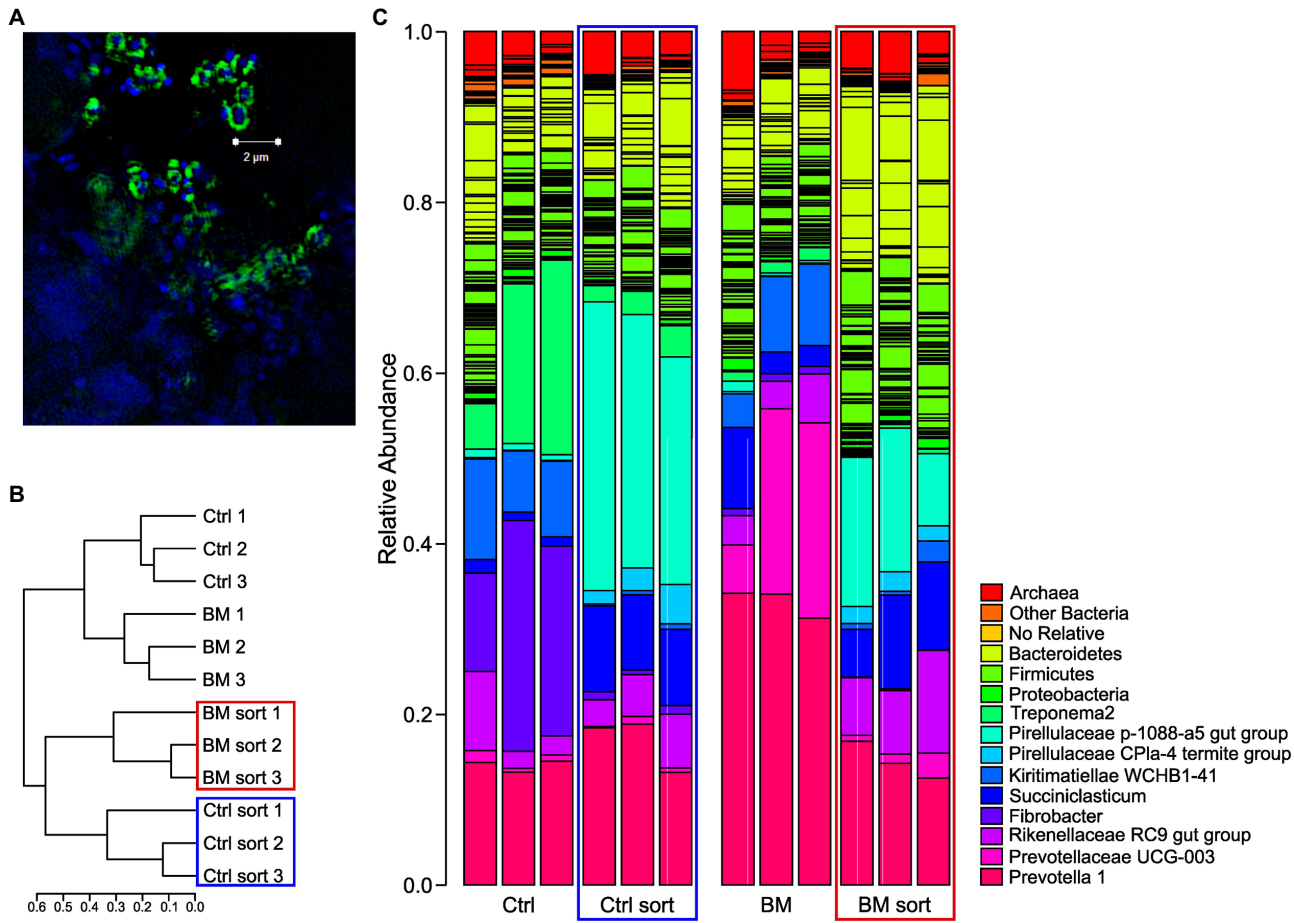
16S rRNA results of rumen communities ± Bio-Mos® before (unsorted) and after (sorted) FACS of FLA-YM incubated communities. **(A)** SR-SIM image of rumen community stained with DAPI (blue) and incubated with FLA-YM (green). **(B)** Phylogenetic tree of sorted and unsorted communities. **(C)** Relative abundance of taxonomic groups in each community. Unsorted control diet = Ctrl, sorted control diet = Ctrl Sort (blue box), unsorted Bio-Mos® diet = BM, and sorted Bio-Mos® diet = BM sort (red box).

To determine if there was a selective prebiotic response to YM by YM-degrading microorganisms, we investigated the changes to the rumen microbial community ± Bio-Mos® using FLA-YM (Figure 1A). After incubating communities ± Bio-Mos® with FLA-YM, cells that showed an interaction with FLA-YM were sorted from the community and quantified by FACS. There was only a small difference in the abundance of FLA-YM uptake in the Bio-Mos® supplemented communities, indicating that there was no unique prebiotic enrichment of YM degrading bacteria over 48 h and that both communities showed the potential to degrade YM. Microbial communities in the control sample displayed an average of 6.87% of cells showing FLA-YM uptake, while communities in the Bio-Mos® treatment samples had 7.67% FLA-YM stained cells (Supplementary Figure 1; Table 1).

**Table 1:** Results of cell sorting of FLA-YM incubated control and Bio-Mos® enriched *in vitro* communities.

Diet	Positive	Negative	Mean FITC	Mean FITC
Control				
Events total	1,000,000	1,000,000		
Cells	97,172	97,522	1,820	436
FLA-YM stained cells	6,674	0	16,867	0
Bio-Mos®				
Events total	1,000,000	1,000,000		
Cells	99,781	99,887	1,813	580
FLA-YM stained cells	7,658	0	12,407	0

### 2.2. FACS of *in vitro* rumen communities

**Figure 2:**
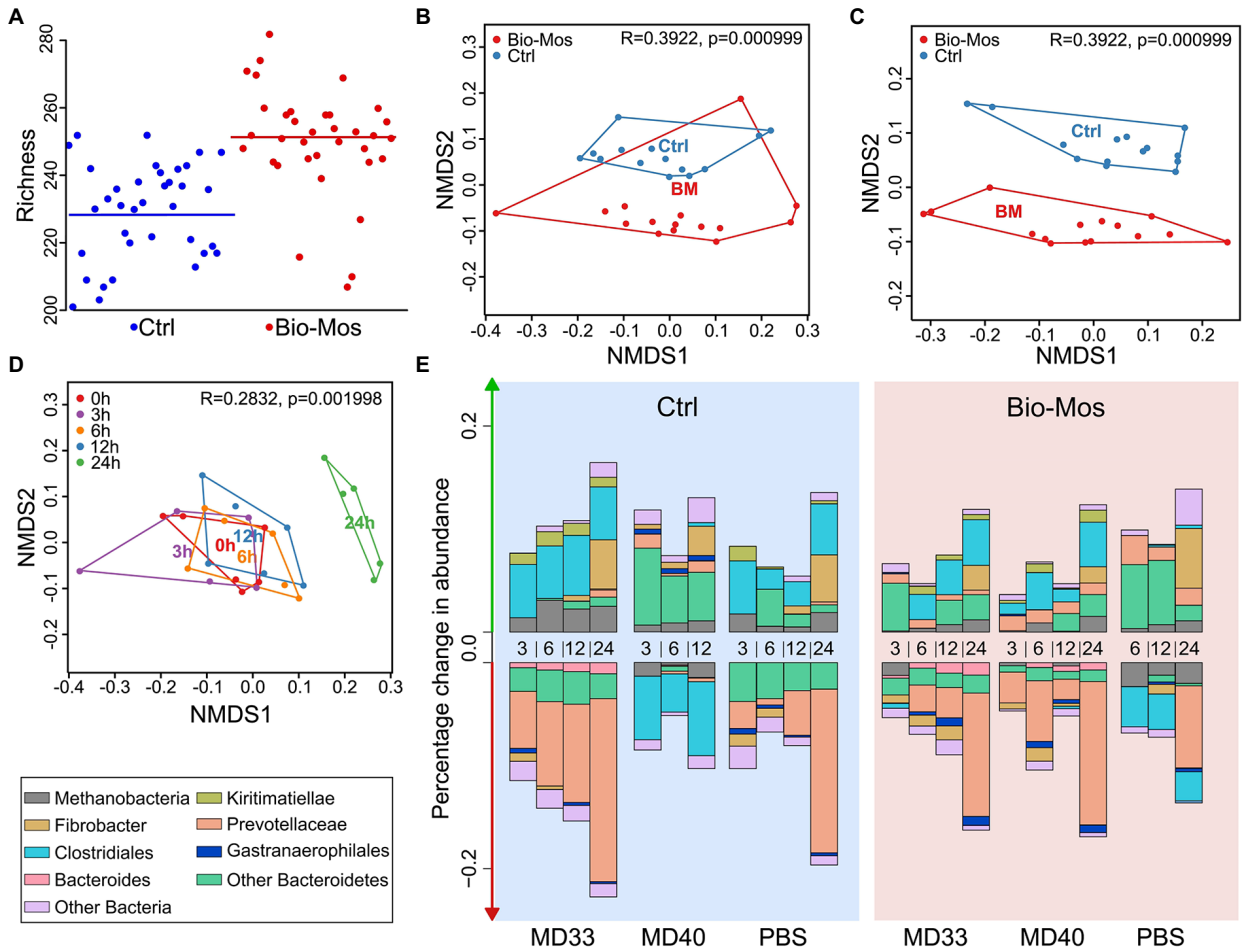
*Ex vivo* community analysis of the prebiotic effect of Bio-Mos®. **(A)** Richness plot showing number of bacterial genera alpha-diversity represented in each sample. **(B)** Non-metric Multi-dimensional Scaling (NMDS) plot comparing microbial communities of rumen samples extracted from cannulated cattle fed Control (blue) or Bio-Mos® (red) diets. There was a significant difference in the microbial community composition between these diets. **(C)** Difference in Bacteroidetes read abundance between rumen Control (blue) and Bio-Mos® (red) rumen communities. **(D)** NMDS plot comparing composition of microbial communities collected from artificial rumen systems at different time points. 0 h in red; 3 h in purple; 6 h in orange; 12 h in blue; and 24 h in green. The composition of the community at 24 h was significantly different than all other time points. 0, 3, 6, and 12 h communities showed no significant difference in their composition. **(E)** Percentage change in abundance of different taxonomic groups in artificial rumen communities. Diet (Control in blue, or Bio-Mos® in red background), Probiotic treatment (MD33, MD40, or control), and sample time point (3, 6, 12, or 24 h) shown on the *x*-axis.

To identify the diversity of YM-interacting organisms between ± Bio-Mos® enrichments, we performed 16S rRNA sequencing after FACS. Cells that showed interactions with YM (Figure 1A; an increased fluorescence signal due to FLA-YM uptake) were sorted (Supplementary Figure 1; Table 1) from the community and identified by 16S rRNA sequencing (Figure 1). The community analysis of the Control and Bio-Mos® treatments showed a significant difference in alpha-diversity, with the Bio-Mos® community showing a higher alpha-diversity (*p* value = 0.077; Figure 2A). The alpha-diversity of the sorted communities was significantly lower (*p* value <0.001) than that of the unsorted samples. Beta-diversity analysis of the communities showed a similar trend, with sorted communities being more similar to each other than the unsorted communities (Figure 1B). ANOSIM analysis of the community dissimilarity showed a significant difference between sorted and unsorted samples (R: 0.822; *p* < 0.001).

Taxonomic analysis of the microbial communities of the two treatments showed that the Control community was dominated by the phyla *Bacteroidetes*, *Firmicutes*, *Fibrobacteres*, *Spirochaetes*, and *Kiritimatiellaeota* (Figure 1C). Bio-Mos® treated samples were composed predominantly of the phyla *Bacteroidetes* and *Kiritimatiellaeota*. Comparatively, the sorted communities of the Control and Bio-Mos® treatments were more similar to each other than to unsorted communities, and were both dominated by *Bacteroidetes*, *Firmicutes*, and *Planctomycetes* (Figures 1B,C). All four communities showed similar abundances of *Rikenellaceae RC9* gut group and *Methanobrevibacter* (Supplementary Figure 2). *Prevotella 1* was highly abundant in all groups, but the most abundant in the unsorted Bio-Mos® community. The Control unsorted and sorted communities showed higher abundance of *Spirochaetaceae treponema 2* than the Bio-Mos® fractions, with the highest abundance seen in the Control unsorted samples. *Fibrobacter* and *Kiritimatiellae WCHB1-41* were also highly abundant in the Control community*. Kiritimatiellae WCHB1-41*, *Prevotellaceae UCG-003*, and *Succiniclasticum* were highly abundant in the Bio-Mos® unsorted communities. *Pirellulaceae 1088-a5* gut group and *Muribacalaceae* were highly abundant in the sorted communities.

### 2.3. Analysis of *ex vivo* rumen batch communities

#### 2.3.1. 16S rRNA sequencing

There were significant changes to the rumen batch culture communities between diets (Figures 2B,C; *p* < 0.001) and time points (Figure 2D; *p* < 0.01). The change with time could also be related to the bacterial inoculation (Figure 2E). Supplementation of the cattle diet with Bio-Mos® changed the richness (Figure 2A) and composition (Figure 2E) of the rumen community. In particular, a shift was observed for Bacteroidetes (Figure 2C). The change in overall microbial composition did not occur within 12 h (measured at 3, 6, 12 h; Figure 2D). However, after 24 h, the composition of the microbial community was significantly different than all other time points. Additionally, a more detailed investigation of taxonomic changes in these treatments over time highlighted some differences for both the prebiotic and bacterial addition, and even further, showed some synbiotic effects (Figure 2E). For example, Bio-Mos® resulted in a decreased percentage change in abundance for *Clostridiales*. Addition of MD40 changed the percent abundance of both *Prevotellaceae* and *Clostridiales* relative to the Control diet PBS and MD33 inoculations. Further, the combination of Bio-Mos® and MD40 resulted in a shift to the change in abundance for *Prevotellaceae*, where the change in abundance went from positive (Control diet) to negative (Bio-Mos® diet), and *Clostridiales*, where the change in abundance went from negative (Control diet) to positive (Bio-Mos® diet).

#### 2.3.2. Composition of indigestible polysaccharide residues

**Figure 3:**
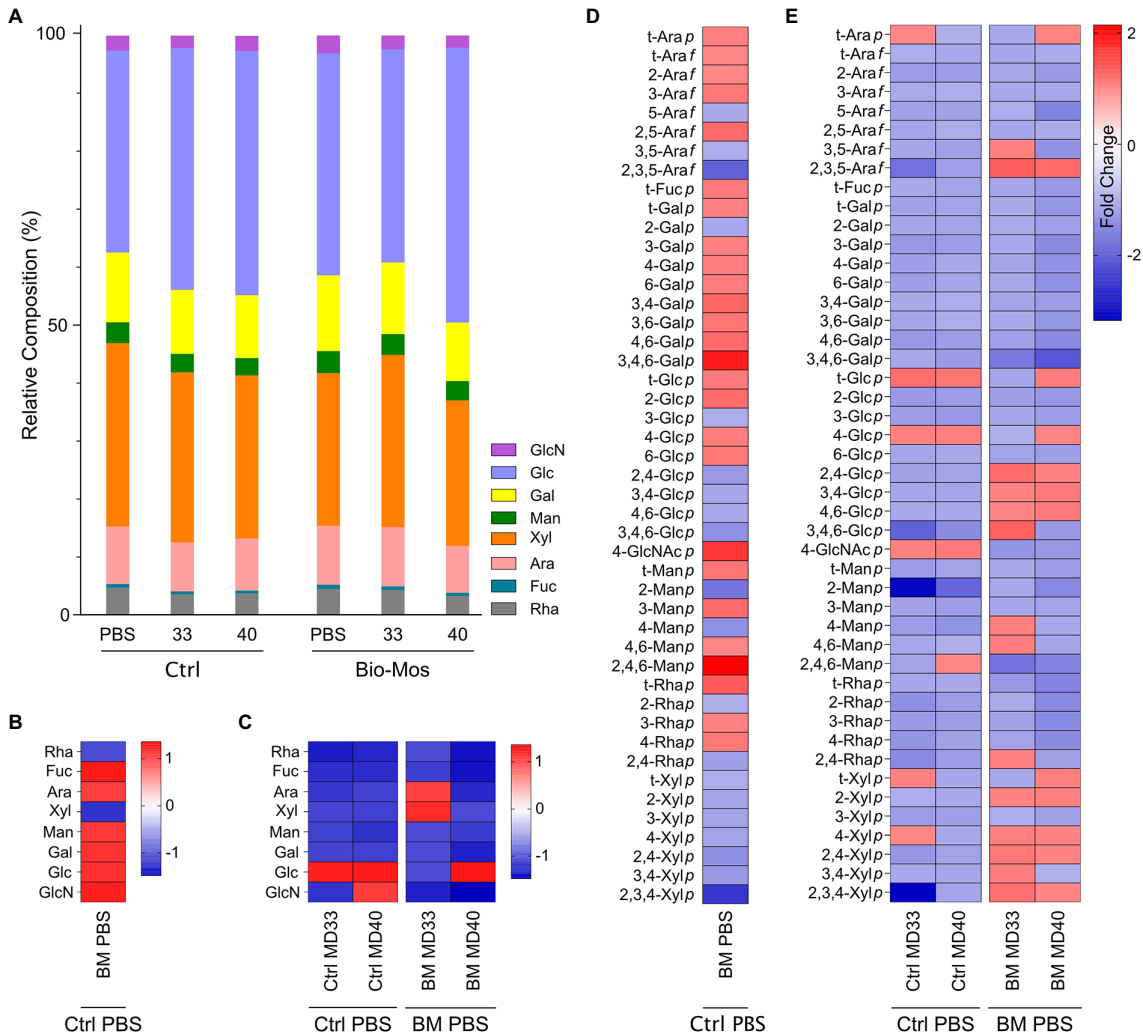
Glycomics analysis of indigestible polysaccharide residue in *ex vivo* rumen communities. **(A)** Non-crystalline cell wall monosaccharide composition. Values are averages from two cows in each diet group, with three separate experiments conducted to rumen fluid collected from each cow. Fold changes in monosaccharide compositions of the **(B)** Bio-Mos® PBS treatment relative to the control PBS treatment and **(C)** Control MD33 or MD40 treatments relative to the control PBS treatment (left) or the Bio-Mos® MD33 or MD40 treatment relative to the Bio-Mos® PBS treatment (right). Values in cells on the map are fold changes in monosaccharide composition between the PBS treatments and any of the five treatment groups (presented as columns) for given monosaccharides (presented as rows), calculated as the ratio of the maximum to the minimum of the linkage values of the two groups. Comparative analysis (heat map) of total cell wall linkages of indigestible residue of **(D)** Bio-Mos® PBS treatment relative to the control PBS treatment and **(E)** Control MD33 or MD40 treatments relative to the control PBS treatment (left) or the Bio-Mos® MD33 or MD40 treatment relative to the Bio-Mos® PBS treatment (right). Values in cells on the map are fold changes in linkage composition between the PBS treatments and any of the five treatment groups (presented as columns) for given linkages (presented as rows), calculated as the ratio of the maximum to the minimum of the linkage values of the two groups. A value with a warm color (positive change number) signifies an increase in the associated monosaccharide or linkage in that sample; a value with a cold color (negative change number) signifies a decrease in the associated monosaccharide or linkage in that sample.

Analysis of monosaccharides and glycosidic linkages was performed to define the glycosidic composition of Bio-Mos® and the base feed, and to detect changes to digestibility of these dietary carbohydrates when administered the prebiotic and/or the *B. theta* strains. Relative composition of monosaccharides released from non-crystalline cell wall fractions of the indigestible polysaccharide residues of different treatment groups are shown in Figure 3A. The dominant monosaccharides are glucose and xylose, with smaller amounts of glucosamine (hydrolysis product of *N*-acetylglucosamine), galactose, mannose, arabinose, fucose, and rhamnose. Fold-change differences in monosaccharide composition include an increase in fucose, arabinose, mannose, galactose, glucose and glucosamine and a decrease in rhamnose and fucose in the Bio-Mos® PBS treatment (Figure 3B). Glucose increased with both MD33 and MD40 inoculation in the absence of Bio-Mos® (Figure 3C). Glucose also increased with MD40 inoculation in the Bio-Mos® treated samples, but decreased with MD33 addition (Figure 3C). Interestingly, arabinose and xylose were the only monosaccharides that increased with MD33 inoculation in the Bio-Mos® samples.

Relative compositions of whole cell wall linkages of the indigestible residues of rumen batch treatments (at the 48 h time point) are shown in Table 2. Comparative analyses (fold changes) of the cell wall linkage compositions of the indigestible residues are shown in Figures 3D,E. The linkages that decrease the most in the Bio-Mos® PBS treatment (Figure 3D) are 2,3,5-Ara*f* and 2,3,4-Xyl*p*; while the linkages that increase the most are 3,4,6-Gal*p*, 4-GlcNAc*p*, and 2,4,6-Man*p*. The linkage analysis of the MD33 and MD40 treatments without Bio-Mos® appear similar with a decreased fold change of most glycosidic linkages (Figure 3E). Key differences between the comparative Control MD33 and MD40 treatments are in the following linkages: t-Ara*p*; 2,3,5-Ara*f*; 3,4,6-Glc*p*; 2-Man*p*; 2,4,6-Man*p*; t-Xyl*p*; 4-Xyl*p*; and 2,3,4-Xyl*p*. The Bio-Mos® MD33 and MD40 treatments when compared to the Bio-Mos® PBS treatment show a similar trend to each other, with small differences (Figure 3E). Comparative linkage composition between MD33 and MD40 treated with Bio-Mos® have different linkages for: t-Arap; 3,5-Ara*p*; 3,4,6-Gal*p*; t-Glc*p*; 4-Glc*p*; 3,4,6-Glc*p*; 4-Man*p*; 4,6-Man*p*; 2,4-Rha*p*; t-Xyl*p*; and 3,4-Xyl*p*.

**Table 2:** Total cell wall linkage composition (%) of indigestible residues of rumen samples from cattle fed diets ± Bio-Mos®.

Diet:	Control			Bio-Mos®		
Linkages	PBS	MD33	MD40	PBS	MD33	MD40
t-Ara*p*	0.1	0.1	0.1	0.1	0.1	0.1
t-Ara*f*	1.3	1.3	1.2	1.4	1.2	1.3
2-Ara*f*	0.9	0.8	0.8	0.9	0.8	0.7
3-Ara*f*	0.4	0.4	0.4	0.5	0.5	0.5
5-Ara*f*	0.6	0.5	0.5	0.5	0.5	0.3
2,5-Ara*f*	0.2	0.2	0.2	0.3	0.3	0.3
3,5-Ara*f*	0.3	0.3	0.2	0.3	0.3	0.2
2,3,5-Araf	0.2	0.1	0.2	0.1	0.2	0.1
t-Fuc*p*	0.2	0.2	0.1	0.2	0.2	0.1
t-Galp	1.4	1.2	1.2	1.5	1.4	1.1
2-Galp	0.2	0.1	0.1	0.2	0.1	0.1
3-Galp	0.3	0.2	0.2	0.3	0.3	0.2
4-Galp	0.3	0.3	0.3	0.4	0.3	0.3
6-Galp	0.6	0.5	0.6	0.7	0.6	0.5
3,4-Galp	0.2	0.2	0.2	0.3	0.2	0.2
3,6-Galp	0.4	0.4	0.4	0.5	0.5	0.4
4,6-Galp	t.r.	t.r.	t.r.	0.1	t.r.	t.r.
3,4,6-Galp	t.r.	t.r.	t.r.	0.1	t.r.	t.r.
t-Glcp	3.3	4.0	3.8	3.7	3.3	4.0
2-Glcp	0.1	0.1	0.1	0.2	0.2	0.1
3-Glcp	0.5	0.5	0.4	0.5	0.5	0.4
4-Glcp	49.5	52.7	53.6	53.9	53.6	55.2
6-Glcp	0.2	0.2	0.2	0.3	0.2	0.2
2,4-Glcp	0.8	0.7	0.7	0.6	0.8	0.6
3,4-Glcp	1.1	1.0	1.0	1.0	1.0	1.1
4,6-Glc*p*	1.6	1.5	1.5	1.5	1.5	1.6
3,4,6-Glc*p*	0.2	0.1	0.1	0.1	0.1	0.1
4-GlcNAc*p*	0.7	0.7	0.8	1.2	0.9	0.9
t-Man*p*	0.2	0.2	0.2	0.2	0.2	0.2
2-Man*p*	0.3	0.1	0.2	0.2	0.2	0.1
3-Man*p*	0.1	0.1	0.1	0.1	0.1	0.1
4-Man*p*	1.2	1.0	0.9	0.9	0.9	0.8
4,6-Man*p*	0.1	0.1	0.1	0.1	0.1	0.1
2,4,6-Man*p*	0.1	0.1	0.1	0.2	0.1	0.1
t-Rha*p*	0.2	0.2	0.2	0.3	0.2	0.2
2-Rha*p*	0.7	0.5	0.5	0.7	0.6	0.5
3-Rha*p*	0.5	0.4	0.4	0.5	0.4	0.4
4-Rha*p*	0.1	0.1	0.1	0.1	0.1	0.1
2,4-Rha*p*	0.4	0.3	0.3	0.3	0.3	0.3
t-Xyl*p*	1.0	1.1	0.9	1.0	0.9	1.0
2-Xyl*p*	1.6	1.5	1.4	1.4	1.4	1.4
3-Xyl*p*	0.4	0.4	0.4	0.4	0.4	0.3
4-Xyl*p*	22	22	20.5	19	20.4	19.8
2,4-Xyl*p*	2.1	1.6	1.8	1.5	1.7	1.5
3,4-Xyl*p*	2.1	2.0	1.9	1.7	1.8	1.7
2,3,4-Xyl*p*	1.0	0.3	0.9	0.4	0.5	0.4

Each value is the average from two cows in a diet group. Three separate experiments were conducted to rumen fluid collected from each cow. t.r. means trace (Mo % < 0.05).

#### 2.3.3. Gas production

**Figure 4:**
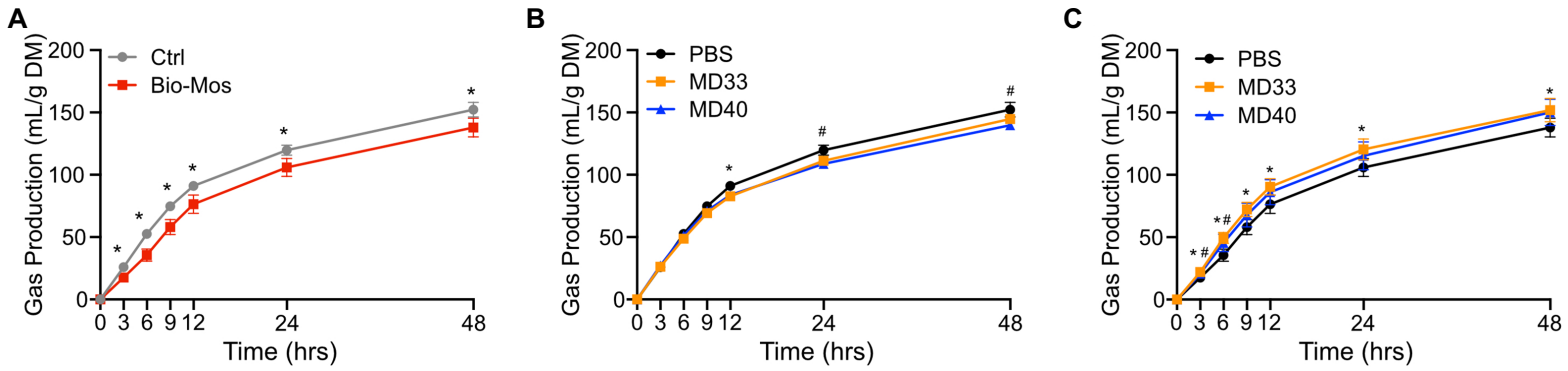
Gas production of *ex vivo* rumen systems. **(A)** Comparing control treatments (PBS added, no probiotic) of Control and Bio-Mos® supplemented diets. *p < 0.05. Comparing differences of high probiotic dose inoculation of MD33 and MD40 strains supplemented with **(B)** Control or **(C)** Bio-Mos®. *p < 0.05 between control and MD33 treatments. ^#^*p* < 0.05 between control and MD40 treatments. *N* = 4. Rumen samples collected from cannulas of cattle fed control or Bio-Mos® supplemented diets.

There were significant differences in gas production of diets ± Bio-Mos® (Figure 4A) at each time point. Rumen samples extracted from cattle fed 1% Bio-Mos® resulted in significantly lower gas production (*p* < 0.05) at each time point sampled, indicating that Bio-Mos® had a significant impact on metabolic processes in the rumen *in vivo*. Bacterial inoculation of rumen samples from cattle not fed Bio-Mos® resulted in few significant changes relative to the PBS treatment (Figure 4B). The gas production decreased significantly (*p* < 0.05) relative to the PBS treatment (Control) at 12 h for the MD33 treatment, and 24 and 48 h for the MD40 treatment. Alternatively, samples from cattle fed a diet supplemented with Bio-Mos® showed significant (*p* < 0.05) increases in gas production relative to the PBS treatment at all time points for the MD33 treatment, and only at 3 and 6 h for the MD40 treatment (Figure 4C). Bacterial inoculation seems to have a more significant effect when the rumen community is exposed to the prebiotic prior to MD33 or MD40 inoculation. This is suggested by the increase in significant differences between the PBS and bacterial treatments, especially MD33, when comparing the rumen samples supplemented with (Figure 4C) or without (Figure 4B) Bio-Mos®.

#### 2.3.4. Volatile fatty acid production

**Figure 5:**
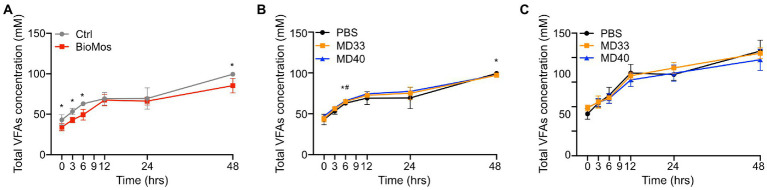
Total volatile fatty acid production of *ex vivo* rumen systems. **(A)** VFA concentrations in control (grey) and Bio-Mos® (red) samples. *Signifies statistical difference between diet samples. **(B)** VFA concentrations of probiotic inoculations of rumen samples taken from cows fed a control diet. **(C)** VFA concentrations of probiotic inoculations of rumen samples taken from cows fed a Bio-Mos® enriched diet. *Signifies statistical difference (*p* < 0.05) between the PBS and MD33 treatments. ^#^Signifies statistical difference (*p* < 0.05) between the PBS and MD40 treatments. *N* = 4.

Supplementing cattle diets with Bio-Mos® showed a significant effect on VFA concentrations over time (Figure 5A; Supplementary Figure 3). However, this depended on VFA and time (Supplementary Figure 3). Butyric acid and valeric acid concentrations were significantly lower in Bio-Mos® supplemented samples at all time points, while there were no significant differences in isovaleric acid concentrations. The levels of C2/C3 were significantly higher in the Bio-Mos® samples at every time point, while total VFA concentration was significantly lower in the Bio-Mos® PBS samples at 0, 3, 6, and 48 h.

Administration of MD33 and MD40 with Bio-Mos® resulted in more changes to VFA concentrations (Figure 5A) than the rumen samples that lacked Bio-Mos® (Figures 5B,C; Supplementary Figure 3). Addition of MD33 resulted in significantly lower concentrations of total VFA, propionic acid, butyric acid, isobutyric acid, isovaleric acid, valeric acid, and caproic acid at the 48 h time point; whereas, MD40 resulted in significantly lower concentrations of propionic acid, butyric acid, isobutyric acid, and caproic acid, and significantly higher levels of C2/C3 at 48 h. There were few significant changes before 48 h.

Addition of bacterial strains with Bio-Mos® resulted in the fewest changes to VFA production (Figure 5C; Supplementary Figure 3). There were no significant changes in total VFA, C2/C3, and acetic acid concentrations. MD33 had significantly lower production of isobutyric acid, isovaleric acid, valeric acid, and caproic acid at the 24 h time point. The MD40 community resulted in significantly lower concentrations of butyric acid, isobutyric acid, isovaleric acid, valeric acid, and caproic acid produced at 24 h.

#### 2.3.5. Ammonia production

**Figure 6:**
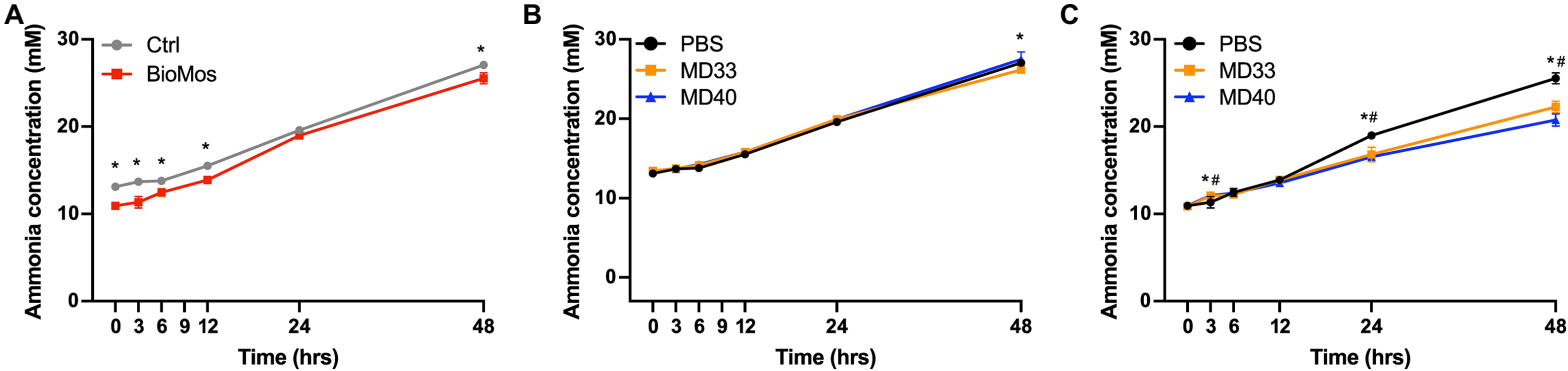
Concentration of ammonia produced in *ex vivo* rumen systems. **(A)** Concentrations produced in control treatments (no probiotic) of samples collected from cattle fed a control diet (grey) or a diet supplemented with 20 g/day Bio-Mos® (red). *Signifies significant difference (*p* < 0.05) between the diet treatments. Samples from cattle fed the **(B)** Control or **(C)** Bio-Mos® supplemented diets were inoculated with PBS (control, black), MD33 (orange), or MD40 (blue). *Signifies statistical difference (*p* < 0.05) between the control and MD33 treatments. ^#^Signifies statistical difference (*p* < 0.05) between the control and MD40 treatments. *N* = 4.

The Bio-Mos® treatment showed significantly lower ammonia production at every time point except 24 h (Figure 6A). The bacterial inoculations of the Control diet samples only resulted in a significant change in the MD33 inoculated samples at the 48 h time point (Figure 6B). In the Bio-Mos® samples, MD33 and MD40 inoculation resulted in significant increased production of ammonia at 3 h, followed by decreased production at 24 and 48 h (Figure 6C).

## 3. Discussion

The rumen microbiome encodes vast catalytic potential to aid in the digestion of complex carbohydrates typically found in a herbivorous diet. The carbohydrate profile of the diet in turn shapes the composition and diversity of the rumen microbiome and drives metabolic processes in the rumen, influencing the amount of VFAs, ammonia, methane, and other metabolites. Forages rich in fiber are often added to grains to offset the high metabolic load from readily available carbohydrates, which are predominantly starches. Other dietary supplements, such as prebiotics and probiotics, have potential to shape the rumen microbiome and mediate metabolic processes to prevent gastrointestinal distress, such as acidosis and liver abscesses. The purpose of this study was to assess the impact of Bio-Mos®, a commercial prebiotic composed of structurally complex carbohydrates, including YM, and bovine-adapted YM-degrading *B. theta* strains on the composition and metabolic processes of the rumen microbiome.

### 3.1. Prebiotic effect

The composition and diversity of the rumen community is known to shift following dietary changes (Pinloche et al., 2013; Matthews et al., 2019), although a core microbiome is maintained (Petri et al., 2013; Henderson et al., 2015). Typically, increased dietary complexity is correlated with increased microbial diversity, although inter-animal variation can also impact diversity (Yang et al., 2018). While our study was limited by the number of cows used as a source of inoculant for the *ex vivo* experiment, we pooled rumen fluid from cows to expand the microbial consortia available for batch cultures. This strategy was successful, as it was observed that Bio-Mos® supplementation resulted in an increase in species richness (Figure 2E) of the rumen microbiome. Although the definition has changed over time (Gibson et al., 2017), a prebiotic was initially defined as “a non-digestible food ingredient that beneficially affects the host by selectively stimulating the growth and/or activity of one or a limited number of bacteria in the colon, and thus improves host health” (Gibson and Roberfroid, 1995). Notably, Bio-Mos® increased the relative abundance of *Bacteroidetes* and *Prevotellaceae* while decreasing *Clostridiales* and *Methanobacteria* (Figures 1C,D), supporting that it has a prebiotic effect on the rumen microbial community. Previously, 6.1 ± 0.5% of cells in complex rumen communities utilized FLA-YM and roughly half of these cells were identified as Bacteroidetes (Klassen et al., 2021). Here, we observed 6.87% of cells showed FLA-YM uptake in our Control sample, with a 0.8% increase in the Bio-Mos® community (Supplementary Figure 1; Table 1). In addition, there was an increase in Bacteroidetes (Figures 1C, 2E), suggesting Bio-Mos® results in the proliferation of bacteria that utilize YM. This can be further seen by the significant decrease in diversity and differences in taxonomic abundances between the FLA-YM sorted vs. Bio-Mos® unsorted communities. However, there is not a direct correlation between groups increasing with Bio-Mos® and those importing FLA-YM, as indicated by the increase in *Pirellulaceae* and *Muribacalaceae* in the sorted communities that was not observed in the Bio-Mos® unsorted samples (Supplementary Figure 2). This result is confounded by the presence of additional yeast cell wall polysaccharides, such as β-1,3-glucans, chitin, and proteins in Bio-Mos® (Klis et al., 2002).

Ruminal carbohydrate metabolism and associated fermentation was significantly altered in cattle fed a Bio-Mos® supplemented diet. Changes in the monosaccharide composition of non-crystalline cell wall polysaccharides (Figure 3B) and the linkage compositions of the whole cell wall polysaccharides (Figure 3D) with Bio-Mos® supplementation were observed and indicate prebiotic-associated shifts in the rumen community that led to proliferation of particular taxonomic groups (Figures 1, 2) and differences in consumption of dietary polysaccharides. Further, Bio-Mos® supplementation resulted in significantly lower production of gas (Figure 4A), total VFAs (Figure 5A), and ammonia (Figure 6A) at almost every time point, indicating decreased microbial fermentation efficiency. Decreased production of VFAs, with the exception of acetate, is known to occur with increased forages in the diet (Wang et al., 2020). Ruminal acidosis occurs due to a build up of organic acids that results when microbial fermentation outweighs utilization by the microbiota and absorption by the host (Nagaraja and Titgemeyer, 2007). Therefore, decreased fermentation efficiency caused by Bio-Mos® could benefit the rumen with a high grain diet or low pH state. The beneficial effects of yeast-based supplements on gastrointestinal function has been previously investigated, however there are mixed results and only a few studies look specifically at beef cattle. Decreased VFA production was observed when supplementing dairy cows with a dry active yeast supplement, although ammonia production did not change (Thrune et al., 2009); whereas, other studies have shown an increase in VFA production when high doses of *Saccharomyces cerevisiae* probiotic were fed to cattle (Pinloche et al., 2013) and dried yeast cell wall extract was given to canines (Van den Abbeele et al., 2020). Although multiple studies have shown increased growth performance of cattle when administered yeast cell wall extracts (Heinrichs et al., 2003; Lei et al., 2013; Broadway et al., 2015), our understanding of the effect of yeast cell wall prebiotics (i.e., Bio-Mos®) on ruminal fermentation *in vivo* is still limited and requires further investigation.

### 3.2. Probiotic effect

Lactic acid bacteria and yeast strains are the most common cattle probiotics, and have been shown to increase cattle immunity and performance and reduce pH levels and scours (Broadway et al., 2015; Uyeno et al., 2015). *Ex vivo* rumen communities were inoculated with one of two *B. theta* strains, MD33 and MD40, previously isolated from the bovine rumen (Klassen et al., 2021). The results indicated some changes to the microbial fermentation pathways; although these changes were less significant than the prebiotic effects of Bio-Mos®. There were some significant differences in total gas (Figure 4B), VFA (Figure 5B), and ammonia (Figure 6B) production with MD33 and MD40 inoculation, however they varied over time. Most of the significant differences occurred at the 48 h time point. This indicated that the bacteria may require a longer period to effect change on the total microbial community. Interestingly, MD33 showed decreased fold changes in glycosidic linkages found in Bio-Mos® relative to MD40, such as 3,4,6-Glc*p* and 2-Man*p* (Figure 3E), suggesting that MD33 addition may target these linkages from other sources in the absence of Bio-Mos®. MD33 was also observed to have a greater effect on total VFA concentration and ammonia production than MD40. In contrast, MD40 significantly reduced gas production at 24 and 48 h time points (Figure 4B); whereas, MD33 did not. Based upon these results, the probiotic effect of MD33 and MD40 is inconclusive. More research is needed to understand the effect of MD33 and MD40 on host health and function of the gastrointestinal tract and to determine if an optimal inoculation dose could have more beneficial outcomes.

### 3.3. Synbiotic effect

There have been very few synbiotic studies performed in ruminant livestock (Markowiak and Śliżewska, 2018). Previously one study in dairy cows showed the combined use of *Lactobacillus casei* and dextrans improved milk production (Yasuda et al., 2007). Here we have combined Bio-Mos® and *B. theta* strains to evaluate their synbiotic potential. MD33 and MD40 strains display differential YM utilization phenotypes; however, both strains contain similar YM targeting PULs (MAN-PULs; Klassen et al., 2021). Despite these similarities at the genetic level, MD33 displayed a more distributive foraging behavior, suggesting YM hydrolysed products could be shared with other microbes in the community. In contrast, MD40 demonstrated a selfish mechanism similar to the well-characterized *B. theta* VPI-5482 type strain (Cuskin et al., 2015). We hypothesized that YM in Bio-Mos® would be selectively metabolized by MD33 and MD40, and stimulate their growth (Markowiak and Śliżewska, 2018). When administered with Bio-Mos®, the effects of MD33 and MD40 on fermentation were increased. MD33 inoculation resulted in changes to key Bio-Mos® linkages relative to MD40, such as decreased t-Glc*p* and 4-Glc*p* and increased 3,4,6-Glc*p*, 4-Man*p*, and 4,6-Man*p* (Figure 3E), suggesting differences in how each *B. theta* strain selectively targets Bio-Mos® carbohydrates. Changes to total gas and ammonia production (Figures 4, 6) suggested there may be a synbiotic effect, while changes to the overall community (Figure 2) and VFA production (Figure 5; Supplementary Figure 3) were less convincing. The effect of MD33 and MD40 was more noticeable when administered with Bio-Mos®; however, it was not clear if the effect of Bio-Mos® was enhanced with MD33 or MD40 inoculation. Although there were no significant changes to the composition of the rumen microbiome with MD33 or MD40 inoculation, the taxanomic changes observed (Figure 2E) suggested that administering Bio-Mos® and YM degrading *B. theta* together as a synbiotic could change how they affect the structure of these complex communities, an effect that could be significant with increased prebiotic and/or inoculation dosage.

## 4. Conclusion

Manipulating rumen fermentation to increase animal performance has been common practice likely since the domestication of ruminants. However, traditional interventions, such as changes to animal feed or metaphylactic administration of antimicrobials, can have detrimental impacts on animal health with broader implications for human health within a one-health context. Understanding ways to improve rumen fermentation to balance animal performance, economic growth, and environmental health is integral for sustainable agriculture. In this regard, developing effective synbiotics requires an understanding of how potential prebiotics and probiotics contribute to the health of the host. Using FLA-PS to study prebiotic interactions with microbial cells can be used to streamline the identification and characterization of prebiotic-bacterial interactions in complex communities and changes to microbial composition, especially when performed together with nutrition studies. Such combinatorial approaches will be integral to decode the complex interactions between feed additives, the rumen microbiome and cattle health to help the cattle industry adapt to changing practices and a sustainable future.

## 5. Materials and methods

### 5.1. FLA-PS incubations and FACS

#### 5.1.1. *In vitro* Bio-Mos® enrichment for FLA-PS incubations

Rumen fluid was collected from one ruminally cannulated cow on a barley silage diet and pooled. Liquid fraction (25 ml) was distributed into vials containing 250 mg barley straw ±2% Bio-Mos® and incubated anaerobically at 37°C. After 48 h incubation, 5 ml of sample was collected for FLA-YM incubation and microscopy (see 5.1.2). An additional 5mL of rumen sample was added to 0.5 ml 100% glycerol, flash frozen, and stored at −80°C until further treatment for FACSeq (see 5.1.3).

#### 5.1.2. Presorted communities

Rumen ± Bio-Mos® enrichment samples were filtered through a cellulose acetate membrane with a 100 μm pore size. One milliliter of filtered rumen sample was added to 1 ml 0.4% FLA-YM or unlabeled YM and incubated anaerobically at 37°C. Samples were collected at 0 (before FLA-YM addition), 25, and 72 h. Cells were fixed in 1% formaldehyde at 4°C overnight, centrifuged (5,000 *× g* for 10 min), and pellets were washed in 1 *×* PBS. The wash step was repeated once more. Samples were stored at 4°C until treated for super-resolution structured illumination microscopy (SR-SIM). Rumen microbiome cells were filtered onto 47 mm, 0.2 μm pore size polycarbonate sterile filters using a gentle vacuum of <200 mbar. The cells were counter stained with 4′,6-diamidino-2-phenylindole (DAPI) and mounted using a Citifluor/VectaShield (4:1) mounting solution. All cells were visualized and enumerated using a fully automated microscope imaging system, as previously described (Bennke et al., 2016), on a Zeiss AxioImager Z2 microscope stand (MicroImaaging GmbH, Carl Zeiss, Germany) with a cooled charged-coupled-device camera (AxioCam Mrm, Carl Zeiss, Germany) and a Colibri LED light source (Carl Zeiss, Germany) with two light emitting diodes (UV-emitting LED, 365 ± 4.5 nm for DAPI and blue emitting LED, 470 ± 14 nm for FLA-PS 488). After image acquisition using the automated system the images were processed using the ACMETOOL3.0 image analysis software (Bennke et al., 2016). All automatic cell counts were validated using manual cell counting.

#### 5.1.3. Super-resolution structured illumination microscopy

Cells were visualized on a Zeiss ELYRA PS.1 (Carl Zeiss, Germany) using 561, 488 and 405 nm lasers and BP 573–613, BP 502–538, and BP 420–480 + LP 750 optical filters. Z-stack images were taken with a Plan-Apochromat 63×/1.4 Oil objective and processed with the software ZEN2011 (Carl Zeiss, Germany).

#### 5.1.4. Fluorescence activated cell sorting

All labeling experiments were performed under anaerobic conditions (87% N_2_, 10% CO_2_ and 3% H_2_). The rumen-glycerol sample (0.1 g) was weighed and diluted it 1:10 g/ml in minimal medium (MM) (6.6 mM KH_2_PO_4_ (pH 7.2), 15 mM NaCl, 100 μM MgCl_2_, 175 μM CaCl_2_, 50 μM MnSO_4_, 5 mM (NH_4_)_2_SO_4_, 15 μM FeSO_4_, 24 μM NaHCO_3_, 1 g/l l-cysteine, 1.9 μM hematin, 6 μM hemin, and 200 ng/ml vitamin B_12_), vortexed thoroughly and centrifuged 3 min at 700 *× g*. The supernatant was kept, and the pellet discarded. The supernatant was then centrifuged 5 min at 6,500 × *g*, the supernatant discarded, and the pellet washed with MM. The pellet was resuspended in 200 μl of MM. FLA-YM was added at a final concentration of 7.6 μg/μl. Incubation was performed at 37°C for 1 h protected from light. The cells were then centrifuged for 5 min at 6,500 × *g*, the supernatant discarded, and the pellet was washed twice with reduced PBS (rPBS). The final pellet was resuspended in 500 μl of rPBS and kept at 4°C protected from light. The bacterial suspension was then diluted in rPBS for flow cytometry analysis. Flow cytometry analysis was performed on a 5 lasers LSR Fortessa, 20 parameters analyzer (BD, United States). Cell sorting was performed on 3 lasers, 13 detector FACSAria-III or 4 lasers, 18-detector FACSAria Fusion (BD, United States).

Data were analyzed using FACSDiva or FlowJo software (BD, United States). To set the thresholds to specifically detect the cells labeled by FLA-YM, we used unlabeled cells as negative Controls. The detection threshold for signals in the fluorescein isothiocyanate (FITC) channel was set at 10^3^ compared to the maximum signal generated by the negative Control at 3 × 10^2^. The gating threshold may be adjusted at different levels to adjust the sensitivity of the assay depending on the probes. 50,000 or 100,000 events per sample were analyzed and FITC fluorescence measured using the FITC channel with excitation at 488 nm and emission at 535 nm. Forward and side scatter gating was performed to exclude doublets. 1 to 3 × 10^6^ cells for each sample were sorted.

#### 5.1.5. 16S rRNA sequencing of sorted and unsorted communities

DNA was extracted from all samples using the AllPrep PowerFecal DNA/RNA kit (Qiagen, Canada) following the manufacturer’s instructions. DNAs were quantified by Qubit Fluorometric Quantification method (Invitrogen, United States). The V4 region (based on *Escherichia coli*) of the 16S ribosomal RNA (rRNA) was targeted for amplification by PCR using the forward primer: 515F GTGCCAGCMGCCGCGGTAA and reverse primer: 806R GGACTACHVGGGTWTCTAAT. The CS1 (ACACTGACGACATGGTTCTACA) and CS2 (TACGGTAGCAGAGACTTGGTCT) tags were used to add a barcode and Illumina adapters. Amplification was performed using Q5 High Fidelity DNA polymerase (BioLabs, New England) with PCR cycles as follows: initial denaturation step of 98°C, for 30 s, before 23 cycles of 98°C, for 10 s, 58°C, for 15 s and 72°C, for 30 s, with the final extension at 72°C, for 2 min. The MiSeq platform was used for 2 *×* 250 bp paired-end sequencing of the resulting PCR products. Sequencing was performed by Génome Québec. Raw sequences were analyzed as below.

### 5.2. Animal sampling and *ex vivo* rumen batch communities

The Canadian Council of Animal Care guidelines were followed to care for the cows used in this study (Olfert et al., 1993), and the project was approved by the Lethbridge Research and Development Centre (ACC#2124).

#### 5.2.1. *Ex vivo* rumen community sampling

Three ruminally cannulated Angus X Hereford crossbred cows were fed 100% (DM basis) alfalfa hay and supplemented with no prebiotic (Control; week one) or 1% Bio-Mos® (treatment; week two) input directly into the rumen. On the seventh day of each treatment, samples were extracted from the rumen 2 h after prebiotic supplementation. To prepare the inoculum, rumen fluid was combined, strained through four layers cheesecloth and transported in anaerobic thermos containers prior to combining with mineral buffer (Wang et al., 2006). Rumen microbial inoculum consisted of 48 ml of a 1:2 (vol:vol) mixture of rumen fluid and mineral buffer and was divided into vials containing 500 mg of the feed ±2% Bio-Mos®. In an anaerobic chamber (atmosphere: 85% N_2_, 10% CO_2_, 5% H_2_ at 37°C), 2 ml of 1 × PBS, or 1.4–1.9 × 10^9^ CFU/ml of MD33 or MD40 were inoculated into seven replicate vials. Four replicate vials were used for sampling at 0, 3, 6, 12, 24, and 48 h and underwent treatment for VFA, ammonium, 16S rRNA sequencing, and glycomics analysis. The other three replicate vials were permanently closed using a rubber stopper and used to measure gas production at 0, 3, 6, 9, 12, 24, and 48 h.

#### 5.2.2. 16S rRNA sequencing analysis of *ex vivo* rumen batch communities

Samples were centrifuged at 20,000 × *g* for 5 min and supernatants were removed. Pellets were flash frozen in liquid nitrogen and stored at −80°C. Pellets were thawed on ice immediately before genomic extractions using a Powersoil kit (Qiagen, Germany). The extracted genomes were sent to McGill GenomeQuebec for Illumina MiSeq PE250 16S rRNA sequencing using the primers 515F – 806R targeting the V4 regions. A total of 3.5 million paired reads were quality trimmed and merged using the BBTools software (Bushnell et al., 2017), resulting in a minimum of 30,000 and maximum of 60,000 per sample. After merging the reads were separated into sample fasta files using the mothur info.fastq command (Schloss et al., 2009). The fasta files were then uploaded to the online classification platform of SILVAngs, which quality trims, clusters, and classifies the reads using the SSU rRNA seed of the SILVA database release 132 (Quast et al., 2013). The output files of the SILVAngs pipeline were then used to analyze and plot microbial community profiles. Community analysis, statistics, and plotting were performed using R (Team, 2020) in R-studio (Team, 2019) with the packages: Phyloseq (McMurdie and Holmes, 2013), picante (Kembel et al., 2010), ggplot2 (Wickham, 2016), and rioja (Juggins, 2019).

All analyses were done on triplicate results of each sample and on an averaged read abundance from the triplicates. There was no significant difference between these analyses. For plotting purposes, only the average analysis was used.

#### 5.2.3. Glycomics analysis of indigestible polysaccharide residue in *ex vivo* rumen batch communities

Each rumen batch community was mixed with 4 volumes of absolute ethanol immediately following its collection. The mixture was vortexed then centrifuged (3,000 × *g*, 30 min). The resulting pellet was collected, washed with methanol, centrifuged to remove the wash (3,000 × *g*, 30 min), then air-dried at room temperature, followed by ball-milling to fine powder using a Mixer Mill MM 200 (Retsch, Germany). Next, alcohol insoluble residue (AIR) was prepared according to the reports (Wood et al., 2018; Low et al., 2020), with slight modifications. Briefly, the sample powder (~30 mg) was soaked (with occasional vortex) three times in 1.5 ml of each of the following solvents including ethyl acetate for 2 h, ethanol/deionized water solution (4:1, v/v) for 8 h, and acetone and methanol (20 min each). After each wash, the sample was centrifuged (3,000 × *g*, 30 min), and the supernatant was discarded. The residue was air-dried then de-starched as previously described (Wood et al., 2018), except that enzymatic hydrolysis of starch was conducted with single enzyme thermostable α-amylase (instead of combined use of α-amylase and amyloglucosidase), the de-starched sample was dialyzed with a molecular weight cut off of 6,000–8,000 Da (instead of 3,500 Da) against deionized water, and the water insoluble residue (instead of all content) in dialysis tubing was collected by centrifugation then vacuum-dried for carbohydrate analysis.

Linkage analysis of the de-starched residue was performed by conversion to their partially methylated alditol acetate (PMAA) derivatives by permethylation, hydrolysis, reduction, and peracetylation followed by GC-MS quantification (Pettolino et al., 2012). Neutral sugar PMAAs were tested on a 7890A-5977B GC-MS system (Agilent, United States) installed with a medium-polarity Supelco SP-2380 column (100 m × 0.25 mm × 0.20 μm, Sigma-Aldrich, United States) with oven temperature programmed to start at 100°C followed by increasing at 1.5°C/min to 220°C, then 1.25°C/min to 250°C (hold 20 min). Amino sugar PMAAs were tested on a Finnigan PolarisQ GC-MS (Thermo Fisher, United States) with a zero-polarity Zebron ZB-1plus column (30 m × 0.25 mm × 0.25 μm, Phenomenex, United States) with optimized oven temperature programmed to start at 55°C (hold 2 min) followed by increasing at 30°C/min to 120°C, 5°C/min to 225°C (hold 5 min), 30°C/min to 260°C (hold 8 min), then 30°C/min to 260°C.

Monosaccharides were released from non-crystalline polysaccharides of the de-starched residue by 2 M trifluoracetic acid (TFA) hydrolysis (at 121°C for 90 min; Foster et al., 2010), converted to alditol acetate (AA) derivatives using an optimized reduction-acetylation procedure (Voiges et al., 2012). The AAs were tested on a 7890A GC-FID system (Agilent, United States) with the above-mentioned Supleco SP-2380 column and an oven temperature program of initializing at 180°C (hold 1 min) then increasing at 3°C/min to 250°C (hold 20 min). Besides, in order for enhanced detection of amino sugars, the AAs were also analyzed by an Agilent 6890 N GC-FID system installed with a zero-polarity Zebron ZB-5MSplus column (60 m × 0.25 mm × 1.0 μm, Phenomenex, United States) with oven temperature programmed to start at 55°C (hold 1 min) followed by increases at 30°C/min to 210°C then 1°C/min to 250°C (hold 15 min). All experiments were conducted in triplicate.

#### 5.2.4. Analysis of *ex vivo* rumen batch Gas production

A Fisherbrand™ Traceable™ Manometer Pressure/Vacuum Gauge (Fisher Scientific, United States) was used to measure gas production by inserting a 22G 1½ needle into the rubber stopper. After each reading, the gauge was disconnected from the needle while in the stopper to vent off remaining gas. Data was plotted in GraphPad Prism version 9.1.0 and statistically analyzed by multiple *t* tests.

#### 5.2.5. Analysis of *ex vivo* rumen batch VFA production

From each treatment vial, 1.5 ml of sample was added to 0.3 ml of 25% metaphosphoric acid on ice, mixed and stored at −20°C until further analysis by gas chromatography (Cottyn and Boucque, 1968; Playne, 1985). Data was plotted in Graph Pad Prism version 9.1.0 and statistically analyzed using multiple t tests.

#### 5.2.6. Analysis of *ex vivo* rumen batch ammonia production

From each treatment vial, 1.5 ml of sample was added to 0.3 ml of 2% sulfuric acid on ice, mixed and stored at −20°C until further analysis using an Astoria-Pacific segmented flow analyzer (Clackamas, OR) (Gentry and Willis, 1988; Rhine et al., 1998). Data was plotted in Graph Pad Prism version 9.1.0 and statistically analyzed using multiple *t* tests.

## Data availability statement

The data presented in the study are deposited in the NCBI repository, BioProject Accession No: PRJNA874102 (https://www.ncbi.nlm.nih.gov/bioproject/PRJNA874102) and PRJNA874103 (https://www.ncbi.nlm.nih.gov/bioproject/PRJNA874103).

## Ethics statement

The animal study was reviewed and approved by Lethbridge Research and Development Centre Animal Care Committee (ACC#2124).

## Author contributions

DA: conceived of the study. LK: FLA-PS preparation and incubations. LJ: animal care and rumen sampling. LK, ML, CA, and TA: rumen batch experiment methodology. LD and BC: FACS. GR and BC: 16S sequencing analysis. XX: monosaccharide and glycosidic linkage analysis of rumen residue. LK: writing – original draft preparation. LK, GR, BC, TA, and DA: writing – review and editing. DA and TA: funding acquisition. All authors contributed to the article and approved the submitted version.

## Funding

Funding for this work was provided by the Beef Cattle Research Council (Project No. FDE.14.17) and the Canadian Institute for Health Research (to BC). GR received funding from the European Union’s Horizon 2020 research and innovation program under the Marie Sklodowska-Curie grant agreement No. 840804. BC holds a tier II Canada Research Chair in Therapeutic Chemistry.

## Conflict of interest

The authors declare that the research was conducted in the absence of any commercial or financial relationships that could be construed as a potential conflict of interest.

## Publisher’s note

All claims expressed in this article are solely those of the authors and do not necessarily represent those of their affiliated organizations, or those of the publisher, the editors and the reviewers. Any product that may be evaluated in this article, or claim that may be made by its manufacturer, is not guaranteed or endorsed by the publisher.
