# Supplementary material for: Fluorescence activated cell sorting and fermentation analysis to study rumen microbiome responses to administered live microbials and yeast cell wall derived prebiotics
Source: Front Microbiol. 2023 Mar 3;13:1020250. doi: 10.3389/fmicb.2022.1020250 (PMC10022430; doi:10.3389/fmicb.2022.1020250)
Supplement: Supplementary file 1 [file Data_Sheet_1.pdf]

Supplementary Material

1 Supplementary Figures

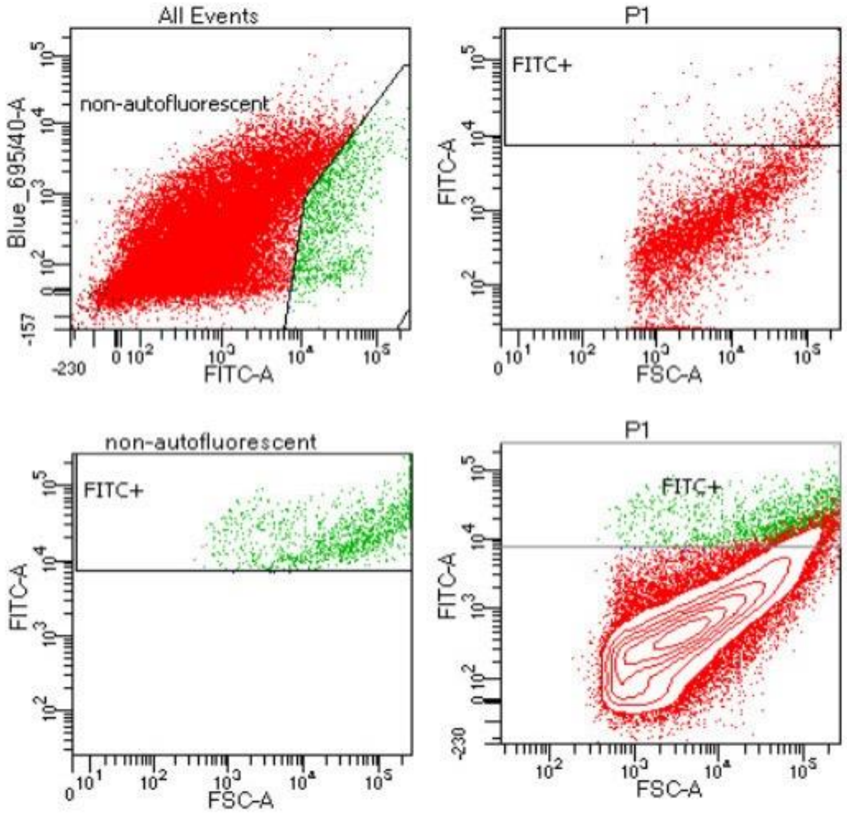

Supplementary Figure 1. Flow plots of rumen communities incubated with FLA-YM and sorted by fluorescence activated cell sorting.

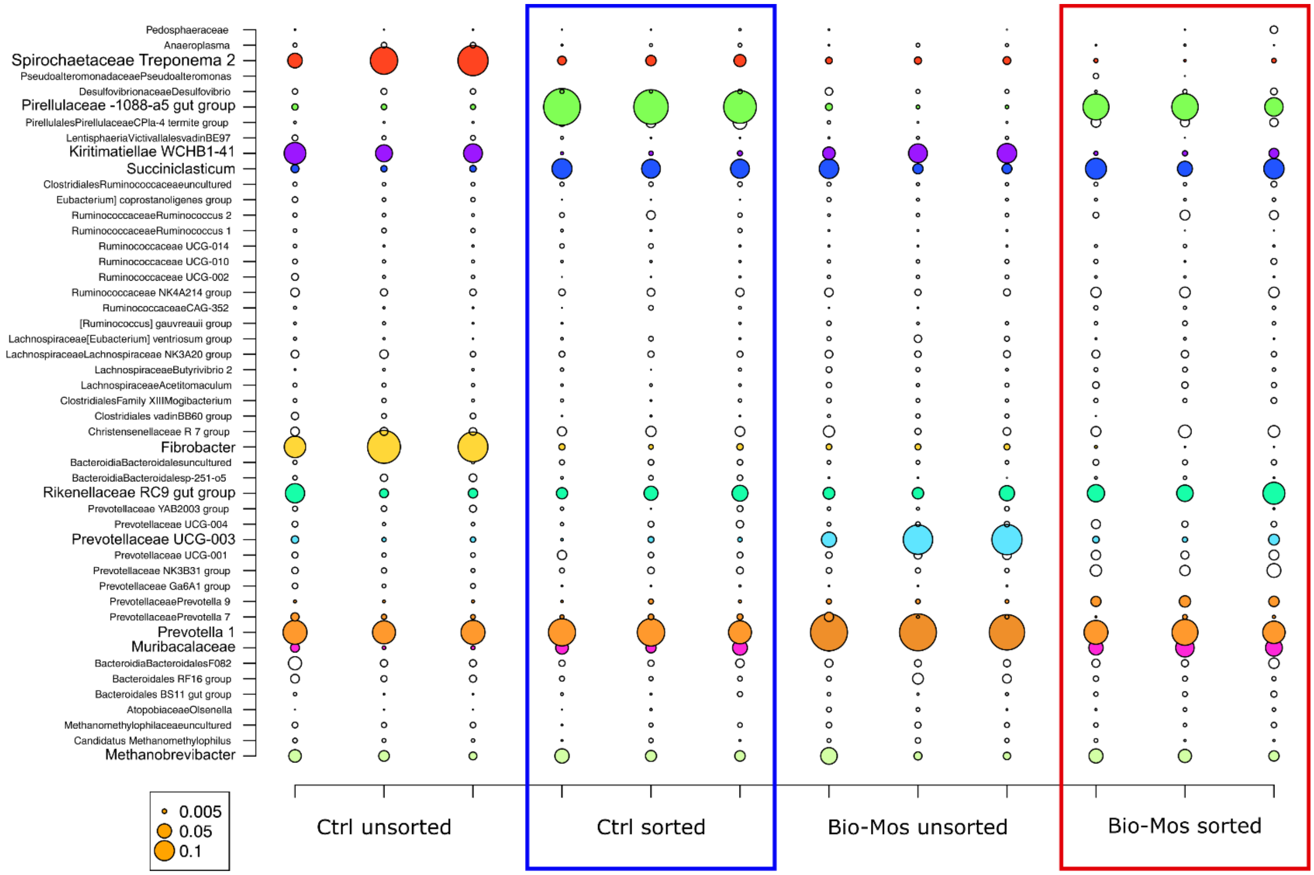

Supplementary Figure 2. Relative abundance of most abundant taxonomic groups of rumen communities ± Bio-Mos® before (unsorted) and after (sorted) FACS of communities incubated with FLA-YM. Unsorted control diet = Ctrl, sorted control diet = Ctrl Sort (blue), unsorted Bio-Mos® diet = BM, and sorted Bio-Mos® diet = Bio-Mos sort (red).

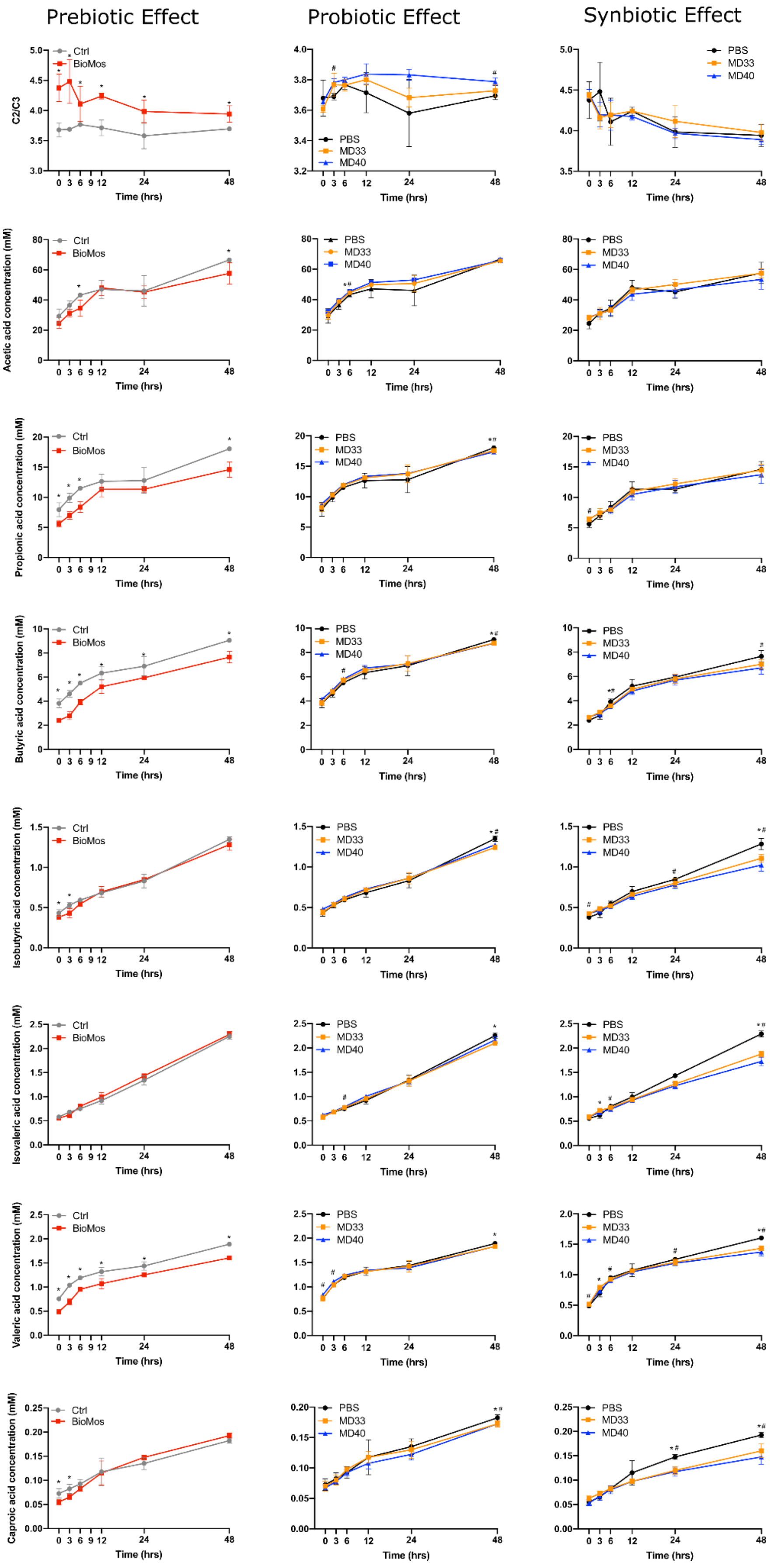

Supplementary Figure 3. Volatile fatty acid production of rumen communities *ex vivo*. Prebiotic effect column compares PBS control sample between Control (grey) and Bio-Mos® (red) diet treatments; * indicates significant differences ($p < 0.05$) between samples. Probiotic effect column compares PBS control (black) to MD33 (orange) or MD40 (blue) inoculated rumen batches of the Control diet. Synbiotic effect column compares PBS control to MD33 and MD40 inoculated rumen batches of the Bio-Mos® diet. Labels on y-axis indicate which VFA concentration is shown in the corresponding graph of that row. * signifies statistical difference ($p < 0.05$) between the control and MD33 treatments. # signifies statistical difference ($p < 0.05$) between the control and MD40 treatments. N = 4.
